# Genes That Extend Lifespan May Do So by Mitigating the Increased Risk of Death Posed by Having Hypertension

**DOI:** 10.1093/ajh/hpad070

**Published:** 2023-08-10

**Authors:** Brian J Morris, Timothy A Donlon

**Affiliations:** Department of Research, NIH Center of Biomedical Research Excellence on Aging, Kuakini Medical Center, Honolulu, Hawaii 96817, USA; Department of Geriatric Medicine, John A. Burns School of Medicine, University of Hawaii, Honolulu, Hawaii 96813, USA; School of Medical Sciences, University of Sydney, Sydney, New South Wales 2006, Australia; Department of Research, NIH Center of Biomedical Research Excellence on Aging, Kuakini Medical Center, Honolulu, Hawaii 96817, USA; Department of Cell and Molecular Biology, John A. Burns School of Medicine, University of Hawaii, Honolulu, Hawaii 96813, USA

**Keywords:** blood pressure, *FLT1*, *FOXO3*, genetics, *GHR*, hypertension, lifespan, *MAP3K5*, mortality, *PIK3R1*, resilience

## Abstract

**BACKGROUND:**

Genetic factors influence lifespan. In humans, there appears to be a particularly strong genetic effect in those aged ≥ 90 years. An important contribution is nutrient sensing genes which confer cell resilience.

**METHODS:**

Our research has been investigating the genetic factors by longitudinal studies of American men of Japanese descent living on the island of Oahu in Hawaii. This cohort began as the Honolulu Heart Program in the mid-1960s and most subjects are now deceased.

**RESULTS:**

We previously discovered various genes containing polymorphisms associated with longevity. In recent investigations of the mechanism involved we found that the longevity genotypes ameliorated the risk of mortality posed by having a cardiometabolic disease (CMD)—most prominently hypertension. For the gene *FOXO3* the protective alleles mitigated the risk of hypertension, coronary heart disease (CHD) and diabetes. For the kinase *MAP3K5* it was hypertension, CHD and diabetes, for the kinase receptor *PIK3R1* hypertension, CHD and stroke, and for the growth hormone receptor gene (*GHR*) and vascular endothelial growth factor receptor 1 gene (*FLT1*), it was nullifying the higher mortality risk posed by hypertension. Subjects with a CMD who had a longevity genotype had similar survival as men without CMD. No variant protected against risk of death from cancer. We have postulated that the longevity-associated genotypes reduced mortality risk by effects on intracellular resilience mechanisms. In a proteomics study, 43 “stress” proteins and associated biological pathways were found to influence the association of *FOXO3* genotype with reduced mortality.

**CONCLUSIONS:**

Our landmark findings indicate how heritable genetic components affect longevity.

## Graphical Abstract

**Graphical Abstract:**
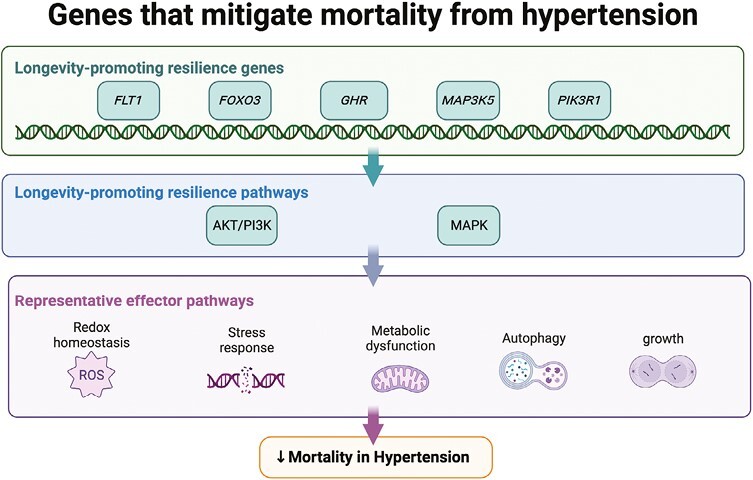

Aging is a consequence of the interplay of chronological age, chronic diseases, lifestyle, and genetic risk. The process may be evident in a primary diseased organ but with time affects multiple other organs which in turn eventually affect multiple other organ systems, ultimately resulting in premature death.^1^ Essential hypertension is a result of arteriolar constriction, and is a well-known risk factor for early death from either myocardial infarction, stroke, heart failure, and kidney disease. Although it is well-known that hypertension can be a consequence of genetic predisposition, we wondered whether there might also be genetic factors that reduce the risk of mortality from having common aging-related conditions such as hypertension.

The present review summarizes the results of our recent studies to investigate the hypothesis that genetic factors might be involved in protection against the premature mortality posed by chronic conditions of aging. Our cohort involved over 8,000 American men of Japanese descent aged 45–68 years recruited in 1965–1968 from World War II Service Records as the Honolulu Heart Program. The participants were then followed up with periodic examinations until the present or death. The cohort became the Kuakini Honolulu-Asia Aging Study with examination 4 in 1991–1993 when the men were 71–93 years old. The examination involved collection of a wide array of numerous demographic and biological parameters as well as blood from each subject. DNA was extracted from the serum buffy coat and examination 4 became the baseline for our genetic studies. The present review will also discuss our recent findings from proteomics studies with BioAge Labs in California investigating the influence of the longevity gene *FOXO3* on proteomic profile and biological pathways involved.

## *FOXO3* (FORKHEAD/WINGED HELIX BOX O MEMBER 3 GENE)

**Figure 1.**
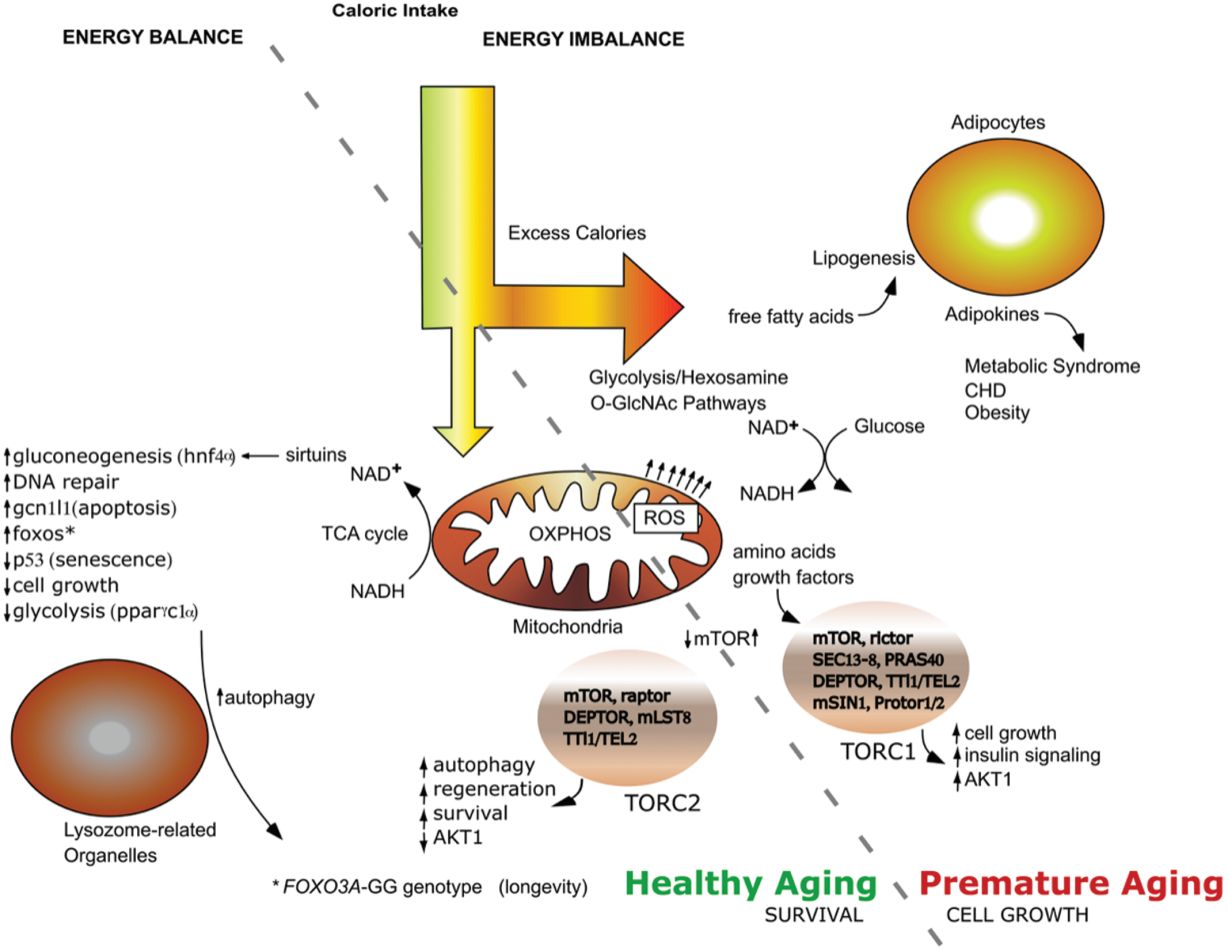
FOXOs are master regulators that translate environmental stimuli arising from insulin, growth factors, nutrients, and oxidative stress into specific gene expression programs. Shown in the diagram are mechanisms by which FoxOs influence healthy aging, in particular several of the key intracellular pathways targeted by FOXO transcription factors. The role of FOXO3 in longevity may involve upregulation of target genes involved in stress resistance, metabolism, cell cycle arrest, and apoptosis. Effective control of FOXO3 in response to environmental stimuli is likely critical to prevent aging and aging-related diseases, including cardiovascular diseases such as hypertension, type 2 diabetes, cancer, and neurodegenerative diseases. The diagram shows how the well-known longevity-associated intervention of caloric restriction helps to maintain the redox state of the cell by cycling calories through the mitochondria so as to restore NAD^+^. Caloric restriction results in activation of sirtuins, leading to activation of FOXO transcription factors, improved autophagy, amino acid recycling via inhibition of mTOR activity, and other mechanisms leading to a healthy aging phenotype. On the other hand, excess calories, particularly from carbohydrates, increase the NADH/NAD^+^ ratio, and results in lipogenesis, overproduction of ROS by mitochondria, poor autophagy, and activation of mTOR as a result of an excess of protein intake. Abbreviations: AKT1, a term derived from the “Ak” mouse strain that develops spontaneous thymic lymphomas (AKT1 is also known as protein kinase B); CAD, coronary artery disease; HNF4α, hepatocyte nuclear factor 4α; GCN1l1, general control of amino acid synthesis 1-like 1; mTOR, mechanistic target of rapamycin; O-GlcNAc, O-linked N-acetylglucosamine; OXPHOS, oxidative phosphorylation; PPARγC1α, peroxisome proliferator activated receptor γ coactivator 1α; TCA, tricarboxylic acid; TORC1, mTOR complex 1; TORC2, mTOR complex 2. This diagram was made by the 2nd author.

Of the many genes implicated in human longevity,^2^ findings for only *APOE*^3^ and *FOXO3*^4^ have been replicated across multiple longevity cohorts in various races and geographic settings globally.^2^*FOXO3* encodes a transcription factor expressed ubiquitously in tissues and it controls pathways implicated in healthy aging and longevity^5^ (Figure 1). The FOXO3 protein binds to specific enhancers in relevant genes, and by recruiting RNA polymerase II modulates gene transcription.^6^*FOXO3* may also function at the genomic level by facilitating long-range gene–gene interactions, causing changes in chromatin conformation and the interaction of topologically associated domains. This results in the regulation of multiple neighboring genes involved in various processes that contribute to cell resilience, namely autophagy, stress response, energy/nutrient sensing, cell proliferation, apoptosis, and stem cell maintenance.^7,8^ We found in those studies that *FOXO3* is positioned at the center of an “aging hub” in which *FOXO3* longevity alleles enhance *FOXO3* expression leading to activation of other genes in its functional neighborhood.

By regulating pathways that suppress vascular smooth muscle cell proliferation and neointimal hyperplasia, the encoded protein (FOXO3 [unitalicized designation]) has a protective effect on vascular aging.^9^ Activation of *FOXO3* transcription in human embryonic stem cells leads to reinforcement of human vascular cell homeostasis, increased resistance to oxidative injury, and retardation of vascular aging.^10^ Loss-of-function studies have shown that FOXO3 helps to maintain homeostasis of a diverse array of vascular cell types.^11,12^

Our research has shown that longevity-associated *FOXO3* single nucleotide polymorphisms (SNPs) comprise a group of variants in close genetic proximity and in linkage disequilibrium, representing a haplotype block.^7^

Previous work by our group has found that longevity-associated genotypes of SNPs were associated with mitigation of the lifespan-shortening effect of having a cardiometabolic disease, which we defined as comprising one or more of the conditions coronary heart disease (CHD), hypertension, and type 2 diabetes in late life. This is an example of resilience, in which *FOXO3* longevity genotypes were not protective *against* these chronic diseases, but, rather, were somehow associated with an extended lifespan, and thus more years of morbidity living with one or more of these conditions.

FOXO proteins play an important role in many cellular processes, including glucose and lipid metabolism, apoptosis, autophagy, cell cycle inhibition, stress resistance, DNA repair, angiogenesis, inflammation, immune response, pluripotency, and differentiation (see review^5^). FOXO transcription factors are thought to protect cells from these insults and assist in repair or elimination of damaged cells.^5^ These functions of FOXO proteins likely underlie their impact on longevity. In contrast, the deregulation of FOXO proteins has been shown to be involved in several diseases owing to their roles in autophagy.^13^ FOXOs are, moreover, tumor suppressors, and regulate multiple crucial intracellular pathways that may contribute to healthy aging.^5^

FOXOs play a key role in cardiovascular disease through the maintenance of cardiomyocyte function in health and pathological conditions such as hyperglycemic and ischemic stress.^14^ FOXO proteins have been shown to inhibit and reverse cardiac hypertrophy, as observed in heart failure, through maintenance of a quiescent state and the promotion of apoptosis after mechanical stress-induced hypertrophy.^15–18^ FOXO proteins are involved in the pathogenesis of type 2 diabetes. The insulin pathway senses the nutritional status of an organism and FOXO transcription factors relay this information to specific transcriptional targets. Therefore, FOXO proteins are considered as metabolic master regulators that control the response to nutrient availability.^19^ FOXO proteins play critical roles in dementia as they are responsible for the maintenance of quiescence of neuronal stem cells and the clearance of reactive oxygen species (ROS).^20^ In a cellular model of Huntington disease, the co-expression of wild-type FOXO1 with mutant huntingtin protein promoted autophagy and clearance of the aberrant protein.^21^ Additionally, levels of nuclear FOXO3 were found to be increased in cells homozygous for Huntington disease mutation.^20^

Our research found that, physiologically, at least one reason for the longer lifespan conferred by *FOXO3* longevity genotype is protection against death from CHD.^22^ We then explored whether this extends to one or more other diseases of aging. In our cohort of American men of Japanese descent, we found that those who had a cardiometabolic disease (hypertension, CHD, and/or diabetes) and one or two alleles of the longevity-associated SNP *rs2802292* lived 19% longer than those who did not have this allele. For hypertension it was 19%, for CHD 17%, and for diabetes 23%.^23^ Although, overall, men with a cardiometabolic disorder did not live as long as men who did not have a cardiometabolic disorder, lifespan of men with a cardiometabolic disorder who had the *FOXO3* longevity genotype did not differ significantly from men without a cardiometabolic disorder.^23^ Furthermore, *FOXO3* genotype had no effect on lifespan of men who did not have a cardiometabolic disorder. These findings led us to conclude that the association of the *FOXO3* longevity genotype with longevity can be attributed to protection against premature mortality in elderly men who have hypertension, CHD, and/or diabetes, that is, who were experiencing cardiometabolic stress. In further research we are investigating other diseases of aging. To date, we have ruled out cancer, but not a genotypic effect of *FOXO3* on stoke and dementia.

## *FOXO3* AND PROTEOMICS

Multiple stress signaling pathways converge on the cell’s protein homeostasis network. Activation of the integrated stress response during aging involves increased expression of stress response genes whose encoded proteins affect pathways capable of slowing the aging processes.^24^ Deregulation in aging-related diseases leads to earlier mortality. From mechanistic, therapeutic, and financial perspectives, it would be valuable to discern pathways that differentiate “healthy” from “unhealthy” aging, namely, avoidance of as compared to resilience against chronic disease. There is abundant literature now that shows changes in serum concentration of particular proteins with aging, and that some of these are proteins associated with increased mortality. A prime aim of GeroScience is to identify biomarkers of aging and implement this knowledge to reduce the burden of aging-related diseases, slow functional decline, and promote healthy aging.^25^ We believe that proteins associated with increased mortality may be used as surrogates/biological markers for life-long stress, permitting us to examine, and perhaps separate, the effects of longevity/resilience genotypes of *FOXO3*, for example, on mortality in individuals experiencing chronic disease-related stress.

In a proteomics study we investigated the biological mechanisms involved in the “mortality resilience” conferred by *FOXO3* longevity-associated genotype. We hypothesized that serum proteins whose levels change with age and are associated with risk of mortality can be considered as “stress proteins.” As a consequence, such proteins may serve as indirect measures of life-long stress. In a collaboration with BioAge Laboratories we quantified 4,500 serum protein aptamers using the Somalogic SomaScan proteomics platform in archived blood samples collected from 995 of our cohort at examination 4 when they were aged 71–83 years. Stress proteins associated with mortality were identified, and age-adjusted multivariable Cox models were used to investigate the interaction of stress proteins with *FOXO3* longevity-associated *rs12212067* genotypes with *P*-values being corrected for multiple comparisons by false discovery rate. This led to the identification of 44 “stress proteins” influencing the association of *FOXO3* genotype with reduced mortality.^26^ The biological pathways identified for these suggested that the *FOXO3* resilience genotype functions by reducing mortality by mechanisms relating to innate immunity, signal transduction, bone morphogenic protein signaling, leukocyte migration, growth factor response, stem cell maintenance, autophagy, and apoptosis.

Based on our findings, we propose a resilience model for *FOXO3* longevity variants in which key factors involved in reducing the mortality include:

1. Response to innate immunity, in which increased cellular stress results in moderation of levels of danger-associated molecular patterns (DAMPs) and pathology-associated molecular patterns (PAMPs).
2. Reduced inflammation, in which the molecules involved serve to initiate formation of inflammasomes and other stress-response structures, resulting in release of inflammatory cytokines and activation of other signaling pathways.
3. Moderation of signal transduction, a process in which signaling pathways (i.e., phosphorylation) mediate responses to damaged organelles, cells, and extracellular structures, and tissues.
4. Moderation of growth factor levels, in which damaged proteins are removed by autophagy and damaged cells are removed by apoptosis, thus increasing levels of growth factors necessary for cellular replacement and tissue repair via stem cell differentiation, without leading to stem-cell exhaustion.
5. Chronic disease, in which chronic disease itself is not delayed, the *FOXO3* resilience genotype delays mortality by extending the functionality of those systems referred to above.

## *MAP3K5* (MITOGEN-ACTIVATED PROTEIN KINASE KINASE KINASE 5 GENE)

We next examined other longevity genes to determine whether they too exerted their effect on lifespan by similar effects. The genes we tested were the human homologs of genes differentially expressed in mouse liver in response to caloric restriction,^27^ an intervention that robustly extends lifespan. We had previously found a number of these were associated with longevity in our cohort.^28^

One was *MAP3K5*, which encodes the kinase, MAP3K5, also termed apoptosis signal-regulating kinase 1 ASK1. MAP3K5 is a member of a family of enzymes involved in kinase signaling cascades. This enzyme mediates signal transduction responses to oxidative stress and inflammation, such as are caused by tumor necrosis factor and lipopolysaccharide. MAP3K5 has a crucial role in the mitochondria-dependent signal transduction pathway activating caspase and leading to apoptosis. This kinase has been implicated in diseases in which ROS and/or endoplasmic reticulum stress are causative factors. It may influence *in vivo* insulin action and obesity, and variants of *MAP3K5* are associated with type 2 diabetes.^29^ MAP3K5 appears to protect against stress-induced disorders and bacterial and viral infection under physiological circumstances. In some pathological conditions, however, MAP3K5 may exert adverse effects by causing excessive cellular apoptosis and increased inflammation, as seen in cardiovascular, neurodegenerative, and inflammatory diseases such as chronic inflammation-induced carcinogenesis.^30^ Its effects involve activation of MKK/JNK and p38 MAPK signal transduction cascades through phosphorylation and activation of several other MAP kinase kinases that in turn activate p38 MAPKs and c-jun N-terminal kinases (JNKs). Both p38 MAPK and JNKs control the transcription factor known as activator protein-1 (AP-1).^31^ The pathways above are critical for senescence, aging, and age-associated cardiovascular diseases. However, MAP3K5 might not cause these, but rather it may be responding to each.^32,33^

We found that in subjects with a cardiometabolic disease, the longevity-associated *C*-allele genotype was associated with a significantly longer lifespan.^34^ Protection against the life-shortening effects of having hypertension was strongest, and against CHD and diabetes was slightly lower. Subjects who did not have one of these diseases outlived men who did. What’s more, *MAP3K5* genotype had no effect on the already longer lifespan of normotensive subjects and those without diabetes or CHD. This led us to suggest that in individuals with one or more of these conditions, having a longevity-associated *MAP3K5* genotype leads to enhancement of resilience mechanisms in cells and tissues to help protect against disease-associated cardiometabolic stress.

## *PIK3R1* (PHOSPHOINOSITIDE-3-KINASE REGULATORY SUBUNIT 5 GENE)

The PI3K family of lipid kinases regulate key intracellular signaling pathways and vesicle trafficking by generating phosphatidylinositol-3,4,5-tris phosphate (PIP_3_).^35^ The eight isoforms of PI3K enzymes comprise three classes. The various members are expressed broadly in tissues and have roles in cardiovascular physiology and cardiovascular disease, with particular involvement in platelet function and thrombosis.^35^ Mutations in the p85α regulatory subunit of PIK3R1 have been implicated in an array of different conditions, one of which is insulin resistance.^36^ In insulin-resistant obese mice, hepatic expression of p85α and the regulatory subunit p55α in liver is decreased.^37^ p85α modulates the cellular response to endoplasmic reticulum (ER) stress by promoting nuclear translocation of X-box binding protein 1 (XBP1) isoform 2 in an ER stress- and/or insulin-dependent manner during metabolic overloading in the liver, and thereby improves glucose tolerance.^38^

In mice, *Pik3r1* is downregulated during caloric restriction.^27^ We found previously that alleles of three adjacent *PIK3R1* SNPs in our cohort were associated with longevity.^28^ In recent research we showed that association of the *TT*/*CC* genotype of our sentinel *PIK3R1* SNP, *rs7709243*, with longevity was due to the ability of this genotype to protect against the increased risk posed by having a cardiovascular disease, defined as hypertension, CHD, and/or cerebrovascular accident (i.e., stroke).^39^ Subjects with the *CT* genotype had a 26% higher risk of dying, whereas *TT*/*CC* subjects exhibited statistically similar lifespan as those without a cardiovascular disease. We found no genotypic effect on lifespan for subjects with diabetes or cancer. We proposed that longevity-associated genetic variants of *PIK3R1* confer cell resilience, thereby mitigating the adverse intracellular effects associated with having a cardiovascular disease.

Interestingly, crosstalk with our FOXO3 findings was apparent, the major transcriptional effector downstream of PI3K being FOXO3.^40^ Inactivation of FoxO (the rodent term) family members in animal models results in suppression of transcriptional programs that control cell proliferation and survival.^41^ FoxO3 in turn upregulates the PIK3 catalytic subunit in response to loss of growth factor signaling.^42^ We found that the three *PIK3R1* SNPs associated with longevity were also associated with body weight, BMI, and forced expiratory volume (FEV1).

## *GHR* (GROWTH HORMONE RECEPTOR GENE)

Low growth hormone (GH) level slows growth, delays maturation, reduces body size, and can attenuate the rate of aging, increase health-span, and extend lifespan.^43^ The effects involve evolutionarily conserved pathways of insulin/insulin-like growth factor and mechanistic target of rapamycin (mTOR), with trade-offs between anabolic processes/growth and lifespan. Thus, the GH deficient Ames dwarf mouse is long lived,^44^ whereas the life span of GH transgenic mice is shortened.^43^ Disruption of the GH receptor (GHR) leads to 55% and 38% longer lifespan in male and female *Ghr*^−/−^ mice, respectively.^45^ Lifespan extension accompanying targeted deletion of both the GH releasing hormone gene and *Ghr*, while increasing lifespan, also leads to a reduction in lean body mass and bone mineral density, accompanied by increased adiposity.^46^ GH deficient dwarf mice are resistant to cancer.^47^ In calorically restricted mice, a well-known model of longevity, *GHR* expression in liver was downregulated 2.1-fold.^27^

In our cohort, shorter stature was associated with greater lifespan.^48^ A case-control study of 13 *GHR* SNPs in men aged ≥ 95 years in our cohort found a significant association of SNP *rs4130113* genotype with longer lifespan in a recessive model.^28^ We then went on to discover that a reason the men lived longer was because the longevity genotype (homozygosity for either the major allele, *A*, or minor allele, *G*, but not heterozygotes, *AG*) was associated with a 17% lower risk of mortality posed by having hypertension.^49^ As was the case for the other longevity genes, hypertensive men with the longevity genotype lived as long as normotensive men. And as with the longevity genes discussed in the previous section, in normotensive men there was no effect of *GHR* genotype on lifespan. No genotypic difference in lifespan was found for diabetes, CHD, and cancer.

## *FLT1* (VASCULAR ENDOTHELIAL GROWTH FACTOR RECEPTOR 1 GENE)

**Figure 2.**
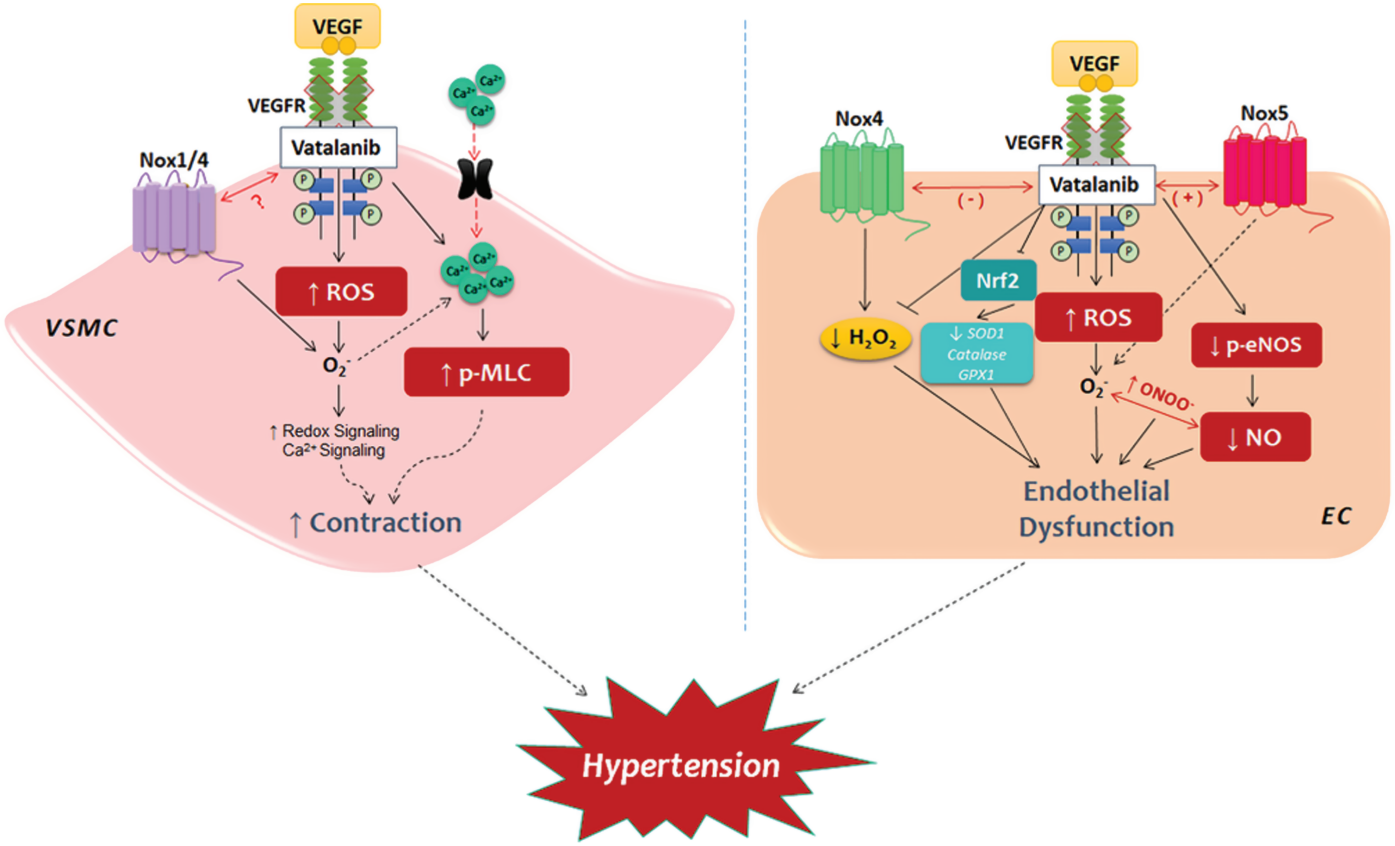
Mechanisms whereby VEGF-A, by binding to the VEGF receptor encoded by *FLT1*, acts on vascular smooth muscle cells to increase vascular resistance and thus blood pressure (left), and causes endothelial dysfunction (right). Each contribute to hypertension. This illustration was originally published in: Neves *et al.* VEGFR (vascular endothelial growth factor receptor) inhibition induces cardiovascular damage via redox-sensitive processes. *Hypertension* 2018; 71(4):638-647. The diagram was obtained from the images website: https://www.bing.com/images/search?q=diagrams%20hypertension%20vascular%20endothelial%20growth%20factor%20receptor&qs=n&form=QBIR&sp=-1&lq=0&pq=diagrams%20hypertension%20vascular%20endothelial%20growth%20factor%20receptor&sc=0-65&cvid=8485BC1F9C3648FBAF66D3A890CE109F&ghsh=0&ghacc=0&first=1 Permission to reuse was sought from the publisher, Walters Kluwer, via the Copyright Clearance Center. The authors paid the publisher US$377.25 to reuse this diagram in the present publication.

*FLT1* encodes the full-length form of vascular endothelial growth factor (VEGF) receptor 1 (VEGFR-1), a cell surface receptor with a ligand-binding region, transmembrane segment, and a cytoplasmic tyrosine kinase domain. Binding of VEGFA and VEGFB to VEGFR-1 regulates cardiovascular and lymphatic blood vessels. This involves cell-type-specific modulation of endothelial cell proliferation. VEGFA and VEGFB contribute to vasculogenesis and angiogenesis to maintain blood supply to tissues. Each counteracts atherosclerosis, CHD, and other adverse cardiovascular conditions.^50^ FLT1 downregulates VEGFA signaling by limiting the availability of free VEGF-A and preventing it from binding to kinase insert domain receptor (KDR/Flk1).^51^ By binding to VEGFR-1 and VEGFR-2, VEGF-A regulates angiogenesis, vascular permeability, and inflammation. VEGF-B binding to VEGFR-1 regulates angiogenesis, redox balance and apoptosis. Figure 2 depicts mechanisms by which VEGFA increases blood pressure.

In essential hypertension, plasma levels of VEGF and sFlt-1 (the soluble form of Flt-1) are elevated.^52^ The increased sFlt-1 might be a response to the elevated blood pressure in hypertension. But because sFlt-1 is anti-angiogenic, it could be causing abnormal angiogenesis, so contributing to hypertension-related complications.^53^

The mouse homolog of the FLT1 gene was amongst a number of genes differentially expressed in calorically restricted mice.^27^ In our cross-sectional study we found that *FLT1* was a longevity gene.^28^ In the longitudinal study of aging and longevity described above we found an association of the major allele of *FLT1* with protection against the risk of mortality posed by hypertension.^54^

## GENE RESILIENCE PATHWAYS

We used an online pathway analysis package^55^ to scan for significant interaction between our longevity genes^56^ (for a brief description of data sources see Mostafavi *et al.*^56^). By default, the GeneMANIA prediction server uses one of two different adaptive network weighting methods. For longer gene lists GeneMANIA adopts a basic weighting method (GeneMANIA^Entry-1^ described in Ref. ^56^, which its website describes as “assigned based on query genes”). This weights each network so that after combining the networks, the query genes interact with each other as much as possible, while interacting as little as possible with genes not in the list. From this analysis we identified involvement of the following pathways:

1. Protein kinase signaling
2. Phosphatidylinositol-mediated signaling
3. Regulation of MAP kinase
4. Insulin signaling
5. Regulation of cell growth/proliferation
6. Regulation of endothelial/epithelial proliferation

## INTERSECTION BETWEEN NUTRIENT-SENSING AND PROTEIN KINASES

Hyperglycemia may be considered one of the quintessential cues for metabolic stress leading to accumulation of advanced glycation end products (AGES) and chronic disease.^57–59^ The various genes listed in the present study were identified through caloric restriction (nutrient deprivation) and are components of a network involved in stress sensing. One can assume that they are also involved in sensing hyperglycemia. This process works by modulation (i.e., phosphorylation) of the insulin, PI3K, and MAPK signaling pathways.

Another pathway that is activated by glucose is the hexosamine biosynthetic pathway (HBP) that results in the addition of O-linked β-*N*-acetylglucosamine (O-GlcNAc) moieties to serine and threonine side chains of amino acids, many of which are in competition to those same sites that are phosphorylated in the above pathways. Approximately 2–5% of intracellular glucose, depending on cell type, enters the HBP, and thus the extent of protein GlcNAcylation is often considered to be sensitive to nutrient (i.e., glucose and/or glucosamine) availability.^60–62^ The HBP is a nutrient sensor that utilizes the donor uridine diphosphate N-acetylglucosamine (UDP-GlcNAc) and integrates nucleotide metabolism (UDP), as well as carbohydrate (Glc), amino acid (N), and fatty acid (Ac) metabolism.^63^ Pathways that are altered by changes in HBP include nutrient sensing, stress response, insulin resistance, leptin levels, diabetes-associated apoptosis, eNOS, leptin levels, and p38 MAP kinase, to name just a few (see Table 1 of Zachara *et al.*^63^).

Angiogenesis is often critical for tumor cells to survive and grow in nutrient-depleted conditions. Akt is activated downstream of VEGF in endothelial cells in the lining of blood vessels, promoting survival and growth. Akt contributes to angiogenesis by activating endothelial nitric oxide synthase (eNOS), and thereby enhancing the production of nitric oxide (NO), which causes vasodilation and vascular remodeling.^64^ Signaling through the PI3K-Akt pathway increases translation of hypoxia-inducible factor-1-α (HIF1α) and HIF2α transcription factors via mTOR^65^ promotes gene expression of VEGF and glycolytic enzymes, allowing metabolism in oxygen-depleted environments.^66^

## LIMITATIONS

**Figure 3.**
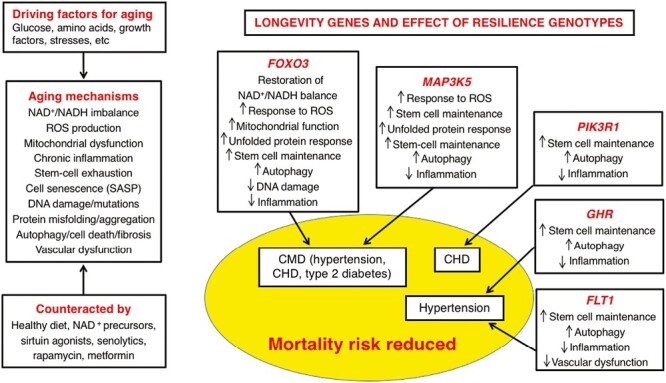
Summary of our findings on the reason longevity-associated genetic variants in particular genes have been found to be associated with longevity.

Because in order to test our hypothesis, the genes we selected from amongst several, *FOXO3*, *MAP3K5*, and *PIK3R1* being the strongest candidates because of strong statistical significance and multiple SNPs associated with longevity, a degree of bias was introduced. In addition, our findings apply to men in the particular racial and demographic population studied (Figure 3).

## FUTURE PERSPECTIVES

Our findings highlight the prospect of interventions to address the absence of longevity genotypes in affected individuals. Short of genetic engineering, future research could investigate therapies that would modulate intracellular pathways that are affected naturally in those who have the longevity genotypes. Irrespective of genetics, already there is a booming industry testing compounds for healthy aging and longevity. Knowledge of genotype, such as revealed in our research, could help tailor advice on use and potential effectiveness of such compounds in individuals interested in improving their health. If they have hypertension or other chronic aging-related conditions, knowledge of a person’s genotype may provide either reassurance of their potential lower risk or a warning of their greater relative risk of mortality.

## SUMMARY

In recent research our group has discovered a possible reason why genes associated with human longevity increase lifespan. In studies of *FOXO3*, *MAP3K5*, *PIK3R1*, *GHR*, and *FLT1* we found that for each, the genetic variants associated with longer lifespan may activate cell resilience mechanisms, leading to amelioration of the adverse effects of hypertension. For *FOXO3*, *MAP3K5*, *PIK3R1*, mitigation of pathological effects of other cardiovascular diseases and/or diabetes represent an additional means whereby individuals with longevity (resilience) genotypes can have a normal lifespan despite possessing one or more of these aging-related conditions. Our review speculates on mechanisms that might be involved. These include autophagy/apoptosis (of damaged cells only), and repair of tissues by a well-maintained reservoir of stem cells. Further research is required to determine whether the concept uncovered by our group may apply to other genes associated with longevity.

## Data Availability

No additional data are available.
